# Evaluation of ketoconazole as a treatment for Cushing’s disease in a retrospective cohort

**DOI:** 10.3389/fendo.2022.1017331

**Published:** 2022-10-07

**Authors:** Camila Viecceli, Ana Carolina Viana Mattos, Maria Carolina Bittencourt Costa, Rafael Borba de Melo, Ticiana da Costa Rodrigues, Mauro Antonio Czepielewski

**Affiliations:** ^1^ Graduate Program in Medical Sciences: Endocrinology, Faculty of Medicine, UFRGS, Porto Alegre, Brazil; ^2^ Endocrinology Division, Hospital de Clínicas de Porto Alegre, UFRGS, Porto Alegre, Brazil; ^3^ Federal University of Rio Grande do Sul (UFRGS), Porto Alegre, Brazil

**Keywords:** Cushing’s disease, Cushing’s syndrome, hypercortisolism, treatment, ketoconazole

## Abstract

**Objective:**

The first-line treatment for Cushing’s disease is transsphenoidal surgery, after which the rates of remission are 60 to 80%, with long-term recurrence of 20 to 30%, even in those with real initial remission. Drug therapies are indicated for patients without initial remission or with surgical contraindications or recurrence, and ketoconazole is one of the main available therapies. The objective of this study was to evaluate the safety profile of and the treatment response to ketoconazole in Cushing’s disease patients followed up at the endocrinology outpatient clinic of a Brazilian university hospital.

**Patients and methods:**

This was a retrospective cohort of Cushing’s disease patients with active hypercortisolism who used ketoconazole at any stage of follow-up. Patients who were followed up for less than 7 days, who did not adhere to treatment, or who were lost to follow-up were excluded.

**Results:**

Of the 172 Cushing’s disease patients who were followed up between 2004 and 2020, 38 received ketoconazole. However, complete data was only available for 33 of these patients. Of these, 26 (78%) underwent transsphenoidal surgery prior to using ketoconazole, five of whom (15%) had also undergone radiotherapy; seven used ketoconazole as a primary treatment. Ketoconazole use ranged from 14 days to 14.5 years. A total of 22 patients had a complete response (66%), three patients had a partial response (9%), and eight patients had no response to treatment (24%), including those who underwent radiotherapy while using ketoconazole. Patients whose hypercortisolism was controlled or partially controlled with ketoconazole had lower baseline 24-h urinary free cortisol levels than the uncontrolled group [times above the upper limit of normal: 0.62 (SD, 0.41) *vs*. 5.3 (SD, 8.21); *p* < 0.005, respectively] in addition to more frequent previous transsphenoidal surgery (*p* < 0.04). The prevalence of uncontrolled patients remained stable over time (approximately 30%) despite ketoconazole dose adjustments or association with other drugs, which had no significant effect. One patient received adjuvant cabergoline from the beginning of the follow-up, and it was prescribed to nine others due to clinical non-response to ketoconazole alone. Ten patients (30%) reported mild adverse effects, such as nausea, vomiting, dizziness, and loss of appetite. Only four patients had serious adverse effects that warranted discontinuation. There were 20 confirmed episodes of hypokalemia among 10/33 patients (30%).

**Conclusion:**

Ketoconazole effectively controlled hypercortisolism in 66% of Cushing’s disease patients, being a relatively safe drug for those without remission after transsphenoidal surgery or whose symptoms must be controlled until a new definitive therapy is carried out. Hypokalemia is a frequent metabolic effect not yet described in other series, which should be monitored during treatment.

## Introduction

Cushing’s disease (CD) results from a pituitary tumor that secretes adrenocorticotropic hormone (ACTH), which leads to chronic hypercortisolism. It is a potentially fatal disease with high morbidity and a mortality rate of up to 3.7 times than that of the general population (1–4) associated to several clinical–metabolic disorders caused by excess cortisol and/or loss of circadian rhythm (5). In general, its management is a challenge even in reference centers (6, 7).

Transsphenoidal surgery (TSS), the treatment of choice for CD, results in short-term remission in 60 to 80% of patients (8). However, recurrence rates of 20 to 30% are found in long-term follow-up, even in those with clear initial remission (9). Drug therapies can help control excess cortisol in patients without initial remission, in cases of recurrence, and in those with contraindications or high initial surgical risk (10).

Nevertheless, specific drugs that act on the pituitary adenoma, which could directly treat excess ACTH, have a limited effect, and only pasireotide is approved for this purpose in Brazil (11, 12). In this scenario, adrenal steroidogenesis blockers are important. One such off-label medication is the antifungal drug ketoconazole, a synthetic imidazole derivative that inhibits the enzymes CYP11A1, CYP17, CYP11B2, and CYP11B1. Because of its hepatotoxicity and the availability of other drugs, it has been withdrawn from the market in several countries (13). In Europe, it is still approved for use in CD, although in the United States, it is recommended for off-label use almost in CD (14–16). Due to the potential benefits for hypercortisolism, ketoconazole has been replaced by levoketoconazole, which the European Union has recently approved for CD with a lower expected hepatotoxicity (17).

Thus, when adrenal inhibitors are used as an alternative treatment for CD, information about the outcomes of drugs such as ketoconazole are important. Clinical studies on these effects in CD are scarce, mostly retrospective, multicenter, or from developed countries (14, 18). A recent meta-analysis on the therapeutic modalities for CD included only four studies (246 patients) that evaluated urinary cortisol response as a treatment outcome and eight studies (366 patients) describing the prevalence of some side effects: change in transaminase activity, digestive symptoms, skin rash, and adrenal insufficiency. Hypokalemia was not mentioned in this meta-analysis (19).

The objective of this study was to evaluate the safety profile of and treatment response to ketoconazole in CD patients followed during a long term in the endocrinology outpatient clinic of a Brazilian university hospital.

## Patients and methods

### Patients

We retrospectively evaluated 38 patients (27 women) diagnosed with CD. These patients, whose treatment included ketoconazole at any time between 2004 and 2020, are part of a prospective cohort series from the Hospital de Clínicas de Porto Alegre neuroendocrinology outpatient clinic.

The diagnostic criteria for hypercortisolism were based on high 24-h urinary free cortisol levels (24-h UFC) in at least two samples, non-suppression of serum cortisol after low-dose dexamethasone testing (>1.8 µg/dl), and/or loss of cortisol rhythm (midnight serum cortisol >7.5 µg/dl or midnight salivary cortisol >0.208 nmol/L). CD was diagnosed by normal or elevated ACTH levels, evidence of pituitary adenoma >0.6 cm on magnetic resonance image (MRI), and ACTH central/periphery gradient on inferior petrosal sinus catheterization when MRI was normal or showed an adenoma <0.6 cm.

CD was considered to be in remission after the improvement of hypercortisolism symptoms or clinical signs of adrenal insufficiency, associated with serum cortisol within reference values, normalization of 24-h UFC and/or serum cortisol <1.8 μg/dl at 8 am after 1 mg dexamethasone overnight, and/or normalization of midnight serum or salivary cortisol. In patients with active disease, to evaluate the ketoconazole treatment response, 24-h UFC was used as a laboratory parameter, as recommended in similar publications (14, 16, 20, 21), but in some cases, we considered elevated late night salivary cortisol and/or 1 mg dexamethasone overnight cortisol (even with normal 24-h UFC), given the greater assessment sensitivity seen through these two methods in the detection of early recurrence when compared with 24-h UFC (22).

#### Inclusion criteria

We included patients with CD and active hypercortisolism who used ketoconazole either as primary treatment, after TSS without hypercortisolism remission, or after a recurrence.

#### Exclusion criteria

We excluded patients with CD and active hypercortisolism who used ketoconazole but had <7 days of follow-up, irregular outpatient follow-up, treatment non-adherence, and incomplete medical records or those who were lost to follow-up.

### Evaluated parameters

Prior to ketoconazole treatment, all patients underwent an assessment of pituitary function and hypercortisolism, including serum cortisol, ACTH, 24-hour UFC, cortisol suppression after 1 mg dexamethasone overnight, midnight serum cortisol, and/or midnight salivary cortisol. The evaluated parameters were sex, age at diagnosis, weight, height, prevalence and severity of hypertension and DM, pituitary tumor characteristics, prior treatment (surgery, radiotherapy, or other medications), symptoms at disease onset, biochemical tests (renal function, hepatic function, and lipid profile), number of medications used to treat associated comorbidities, data on medication tolerance, and reasons for discontinuation, when necessary.

The clinical parameters observed during treatment were control of blood pressure and hyperglycemia, anthropometric measurements (weight, height, and body mass index), jaundice, and any other symptoms or adverse effects reported by patients.

The biochemical evaluation included fasting glucose, glycated hemoglobin, lipid profile (total cholesterol, high-density lipoprotein, low-density lipoprotein, and triglycerides), markers of liver damage (transaminases, bilirubin, gamma-glutamyl transferase, and alkaline phosphatase), electrolytes (sodium and potassium), and renal function (creatinine and urea). Hypecortisolism was accessed preferentially by 24-h UFC, however, late-night salivary cortisol and cortisol after 1 mg overnight dexamethasone could also be used.

### Study design

This retrospective cohort study included patients with CD who were followed up at the Hospital de Clínicas de Porto Alegre Endocrinology Division, with their medical records from the first outpatient visit and throughout clinical follow-up collected. This study was approved by the Hospital de Clínicas de Porto Alegre Research Ethics Committee (number 74555617.0.0000.5327).

### Outcomes

Hypercortisolism was considered controlled when the 24-h UFC and/or late-night salivary cortisol (LNSC) and/or overnight 1 mg dexamethasone suppression test (DST) levels were normalized in at least two consecutive assessments. Hypercortisolism was considered partially controlled when there was a 50% over-reduction in 24-h UFC and/or LNSC and/or DST levels but still above normal. A reduction lower than 50% in these parameters was considered as non-response.

We also assessed the ketoconazole doses that resulted in 24-h UFC normalization, maximum dose, medication tolerance, adverse effects, and changes in liver, kidney, and biochemical function. Due to the characteristics of this study, these outcomes were periodically evaluated in all patient consultations, which occurred usually every 2 to 4 months.

### Data collection

This retrospective cohort evaluated outpatient medical records and any tests indicated by the attending physician as a pragmatic study. Ketoconazole use followed the department’s care protocol, which is based on national and international guidelines (4), and all patients received a similar care routine: the recommended initial prescription was generally taken in two to six doses at 100 to 300 mg/day. It was then increased by 200 mg every 2 to 4 months until hypercortisolism was controlled or side effects developed, especially those related to liver function. The maximum prescription was 1,200 mg/day. Clinical follow-up of these patients was performed 30 days after starting the medication and every 2–4 months thereafter (23).

Clinical, anthropometric, laboratory, and other exam data were collected through a review of the hospital’s electronic medical records for the entire follow-up period. Data from the first and last consultation were considered in the final analysis of all parameters.

### Statistical analysis

Baseline population characteristics were described as mean and standard deviation (SD) or median with interquartile ranges (25–75) for continuous variables. The chi-square test was used to compare qualitative variables, and Student’s *t*-test or ANOVA was used to compare the quantitative variables. The Mann–Whitney *U*-test was used for unpaired data. *P*-values <0.05 were considered significant. Statistical analysis was performed in SPSS 18.0 (SPSS Inc., Chicago, IL, USA) and R package geepack 1.3-1.

## Results

Treatment with ketoconazole was indicated for 41 of the 172 CD patients. In 3/41 patients, ketoconazole was unallowed due to concomitant liver disease, and 38 received ketoconazole during CD treatment between 2004 and 2020. Of these, five were excluded due to insufficient data to determine the response to ketoconazole (short treatment time, irregular follow-up, incomplete medical records, or lost to follow-up). The baseline characteristics of every sample are shown in **Table 1**. Thus, 33/41 patients were included in the final analysis. The patients were predominantly women (84.2%) and white (89.5%); 11 had microadenoma, 15 had macroadenoma, and 11 had no adenoma visualized. In 12/33 patients, pituitary imaging was not performed immediately before starting ketoconazole. Hypertension was observed in 26 patients (78%) and DM in 12 patients (36%). The mean age at CD diagnosis was 31.7 years.

**Table 1:** Baseline clinical data of Cushing’s disease patients treated with ketoconazole.

Variable	Total, *n* = 38 (%)
Sex (F/M)	32/6 (84.2/15.8%)
Age (years)	31.7 (11–51)
Ethnicity (White/non-White)	34/4 (89.5/10.5%)
Facial plethora	22 (57.9%)
Diabetes mellitus	12 (36%)
Hypertension	26 (78%)
Purpuric striae	21 (55.3%)
Acne	11 (28.9%)
Proximal weakness	19 (50%)
Supraclavicular fat	17 (44.7%)
“Buffalo hump”	22 (57.9%)
Psychiatric illness	12 (31.6%)
Microadenoma Macroadenoma Normal MRI Unavailable	11 (28.94%) 15 (39.47%) 11 (28.94%) 1 (2.63%)

**Figure 1:**
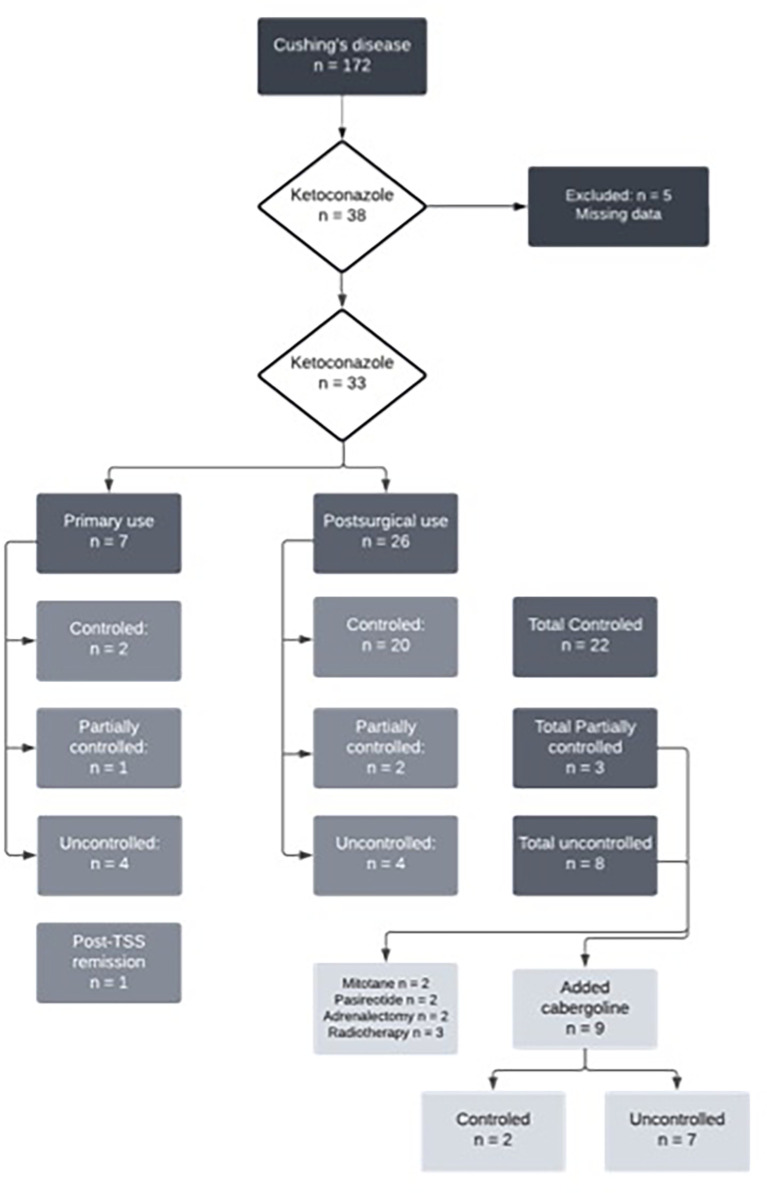
Flowchart of ketoconazole treatment in Cushing's disease patients.

Of the 33 patients with complete data, 26 (78%) underwent TSS prior to starting ketoconazole, five of whom (15%) had also undergone radiotherapy. Thus, seven patients used ketoconazole as primary treatment since performing a surgical procedure was impossible at that time. Of these, four had no response to ketoconazole, one had a partial response, and two had a complete response. At follow-up, four of these patients underwent their first TSS, and three continued the ketoconazole therapy, achieving full UFC control. Among those who used ketoconazole after TSS (*n* = 26), 20 had a complete response, two had a partial response, and four had no response. **Figure 1** shows the study flow chart and patient distribution throughout the treatment.

Individual patient data are described in **Table 2**. The duration of ketoconazole use ranged from 14 days (in one patient who used it pre-TSS) to 14.5 years. The total follow-up time of the 22 patients with controlled CD ranged from 3 months to 14.5 years, with a mean of 5.33 years and a median of 4.8 years.

**Table 2:** Individual data.

ID	Sex	Time(months)	Prior TSS	Prior RDT	Dose variation (mg)^a^	Daily doses	Baseline ACTH	Baseline X > ULNUFC	Final X > ULNUFC	Final therapeutic response	Comorbidities	Altered liver function	Regular use	Intolerance/adverse effects	Outcome
1	F	42	Yes	No	400–600	3	41	0.13	0.4^b^	Complete	SAH and DM	No	Yes	No	Continued use
2	F	7	Yes	Yes	100–200	2	88	1	0.79^b^	Complete	SAH	No	Yes	Yes, nausea	Interrupted due to intolerance
3	F	5	No, pre-TSS use	No	200–400	2		5.4	0.39	Partial	SAH	Yes—drug hepatitis	Yes	Yes, hepatitis	Interrupted due to drug hepatitis
4	F	120	No	No	200–800	4	76	2.92	0.8^b^	Complete	–	No	Yes	Yes	Pseudotumor cerebri resolved with acetazolamide
5	F	126	Yes	No	150–1,000	4 to 5	129	0.05	-0.36	Complete	Dyslipidemia	No	Yes	No	Continued use
6	F	90	Yes	No	300–1,000	4 to 5	26	3.9	-0.7	Complete	SAH and DM	No	Yes	No	Underwent radiotherapy
7	F	63	Yes	No	400–1,000	3	148	5.8	-0.46	Complete	SAH	No	Yes	No	Underwent radiotherapy
8	F	26	No, pre-TSS use	No	600–800	4	106	2.56	0.39^b^	No response	SAH and DM	No	Yes	No	Underwent TSS
9	F	81	Yes	No	600–1,200	5	24	22.2	5.68^b^	No response	SAH	No	Yes	Yes	Underwent bilateral adrenalectomy
10	F	86	Yes	No	400–1,200	4 to 5	65	0.72	-0.47	Complete	–	Important and isolated elevation of GGT	Yes	No	Underwent new TSS
11	F	51	Yes	No	400–800	2	48	-0.68^b^	-0.8	Complete	SAH and dyslipidemia	Discrete and isolated elevation of GGT	Yes	No	Continued use
12	F	14 days	No	No	600	3	121	4.3	3.5	No response	SAH and DM	No	Yes	No	Underwent TSS	
13	F	15	Yes	No	600–800	4	50	0.1	-0.06	Complete	SAH, DM and dyslipidemia	Discrete and isolated elevation of GGT	No	No	Continued use	
14	F	85	Yes	No	200–800	4	37	2.11	-0.38^b^	No response	SAH	Elevation of TGO and GPT, discontinued medication	Yes	Yes	Underwent radiotherapy	
15	F	119	Yes	No	600–1,200	4 to 5	40	2.31	1.3	Partial	SAH, DM and dyslipidemia	Discrete and isolated elevation of GGT	Yes	No	Underwent radiotherapy	
16	F	14	Yes	Yes	600	3	300	0.27	-0.06^b^	Partial	SAH and dyslipidemia	No	Yes	No	Underwent bilateral adrenalectomy	
17	M	29	Yes	No	400–600	3	54	1.62	-0.6	Complete	–	No	Yes	Yes, loss of appetite	Ketoconazole suspended due to loss of appetite	
18	F	5	Yes	No	400	2	65	- 0.08^b^	-0.8	Complete	SAH and DM	Discrete and isolated GGT uptake	Yes	No	Underwent radiosurgery	
19	M	42	No, pre-TSS use	No	400–1,200	2 to 6	51	3.67	2.8	No response	SAH	Discrete elevation of TGO and GGT	No	No	Underwent TSS	
20	F	6	Yes	No	400	2	115	3.9	2^b^	No response	–	No	Yes	No	Lost to follow-up
21	F	125	Yes	Yes	400–1,000	3 to 5	84	1.34	-0.02	Complete	SAH	No	Yes	Yes, diarrhea	Underwent bilateral adrenalectomy
22	M	34	Yes	Yes	100	2		0.3	-0.8	Complete	SAH, DM and dyslipidemia	No	Yes	No	Lost to follow-up
23	F	53	Yes	No	200–600	2 to 4	61	1.19	-0.6	Complete	SAH and DM	No	Yes	Yes, dizziness	Continued use
24	M	3	Yes	No	600	3	1,696	34	0.8^b^	Complete	SAH	No	Yes	No	Underwent bilateral adrenalectomy
25	F	133	Yes	No	400	2	89	0.4	-0.8	Complete	SAH	No	Yes	No	Continued use
26	F	23	Yes	No	200–800	2 to 4	57	0.53	-0.08	No response	–	No	Yes	No	Underwent radiosurgery
27	F	38	No, pre-TSS use	No	400–600	2 to 3	102	4.1	-0.3	Complete	SAH and DM	No	Yes	Yes, dizziness and loss of appetite	Continued use
28	M	111	Yes	No	400—1,200	3 to 4	75	3.9	-0.8	Complete	SAH	Discrete elevation of TGO, GPT, and GGT	Yes	Yes, nausea	Underwent radiotherapy
29	F	8	No, pre-TSS use	No	300	3	92	2.2	0.8^b^	No response	SAH	No	Yes	No	Underwent TSS
30	F	132	Yes	No	200–400	2 to 3	20	-0.2^b^	-0.5	Complete	SAH, DM and dyslipidemia	No	Yes	No	Continued use
31	F	40	Yes	No	400–800	2 to 3	21	0.7	-0.99	Complete	SAH and DM	No	Yes	No	Continued use
32	F	64	Yes	No	400–800	2 to 4	41	-0.7 ^b^	-0.6	Complete	–	Discrete and isolated elevation of GGT	Yes	No	Underwent radiotherapy
33	F	173	Yes	Yes	400–800	2 to 4	40	0.05	-0.5	Complete	SAH	No	No	No	Pregnancy continued using ketoconazole without complications

RDT, radiotherapy; DM, diabetes mellitus; GGT, gamma glutamyl transferase; SAH, systemic arterial hypertension; TGO, oxaloacetic transaminase; GPT, glutamate–pyruvate transaminase; TSS, transsphenoidal surgery; ULN, upper limit of normal.

a The highest dose used was not necessarily the final dose used.

b In these patients, the criterion used to consider active hypercortisolism was late night salivary cortisol and/or elevated 1 mg dexamethasone overnight.

### Therapeutic response

Relative therapeutic response data are described in **Table 3**. Patients whose hypercortisolism was controlled or partially controlled with ketoconazole had lower baseline 24-h UFC than the uncontrolled group [times above the upper limit of normal: 0.62 (SD, 0.41) *vs*. 5.3 (SD, 8.21); *p* < 0.005, respectively], in addition to more frequent prior TSS (*p* < 0.04). In some patients (4/33), 24-h UFC was in the normal range at the beginning of ketoconazole therapy, but they were prescribed with the medication due to the clinical recurrence of CD associated to cortisol non-suppression after 1 mg dexamethasone overnight and/or abnormal midnight salivary or serum cortisol.

**Table 3:** Baseline characteristics of Cushing’s disease patients according to therapeutic response to ketoconazole.

	Controlled/partially controlled		Uncontrolled	*p*
Sex (F/M)	17/8 (68/32%)	7/1 (87.5/12.5%)		0.2281
Initial UFC (times > ULN)	0.62 ± 0.41 (-0.71–0.73)	5.3 ± 8.21 (-0.14–34)		< 0.005
Final UFC (times > ULN)	-0.48 ± 0.55 (-0.99–1.3)	5.13 ± 6.58 (-0.09–19.9)		< 0.005
ACTH	83.6 ± 81.8 (20–300)	172.3 ± 408.2 (1–1696)		0.635
Basal cortisol	18.8 ± 9.5 (8.1–43)	26.5 ± 12.39 (5.8–47)		0.092
BMI	34.5 ± 7.6 (25.8–48)	31.3 ± 6.5 (23.6–50.4)		0.268
Final dose	468 ± 314 (0–1,000)	562 ± 184 (300–800)		0.576
Fasting blood glucose	111.5 ± 39.9 (69–177)	120.7 ± 46.4 (77–219)		0.881
Prior TSS (Y/N)	22/3 (88/12%)	4/4 (50/50%)		0.043
RDT (Y/N)	9/16 (36/64%)	2/6 (25/75%)		0.56
Regular use (Y/N)	23/2 (92/8%)	6/2 (75/25%)		0.2

ACTH, adrenocorticotropic hormone; TSS, transsphenoidal surgery; RDT, radiotherapy; UFC, urinary free cortisol; ULN, upper limit of normal.

**Figure 2:**
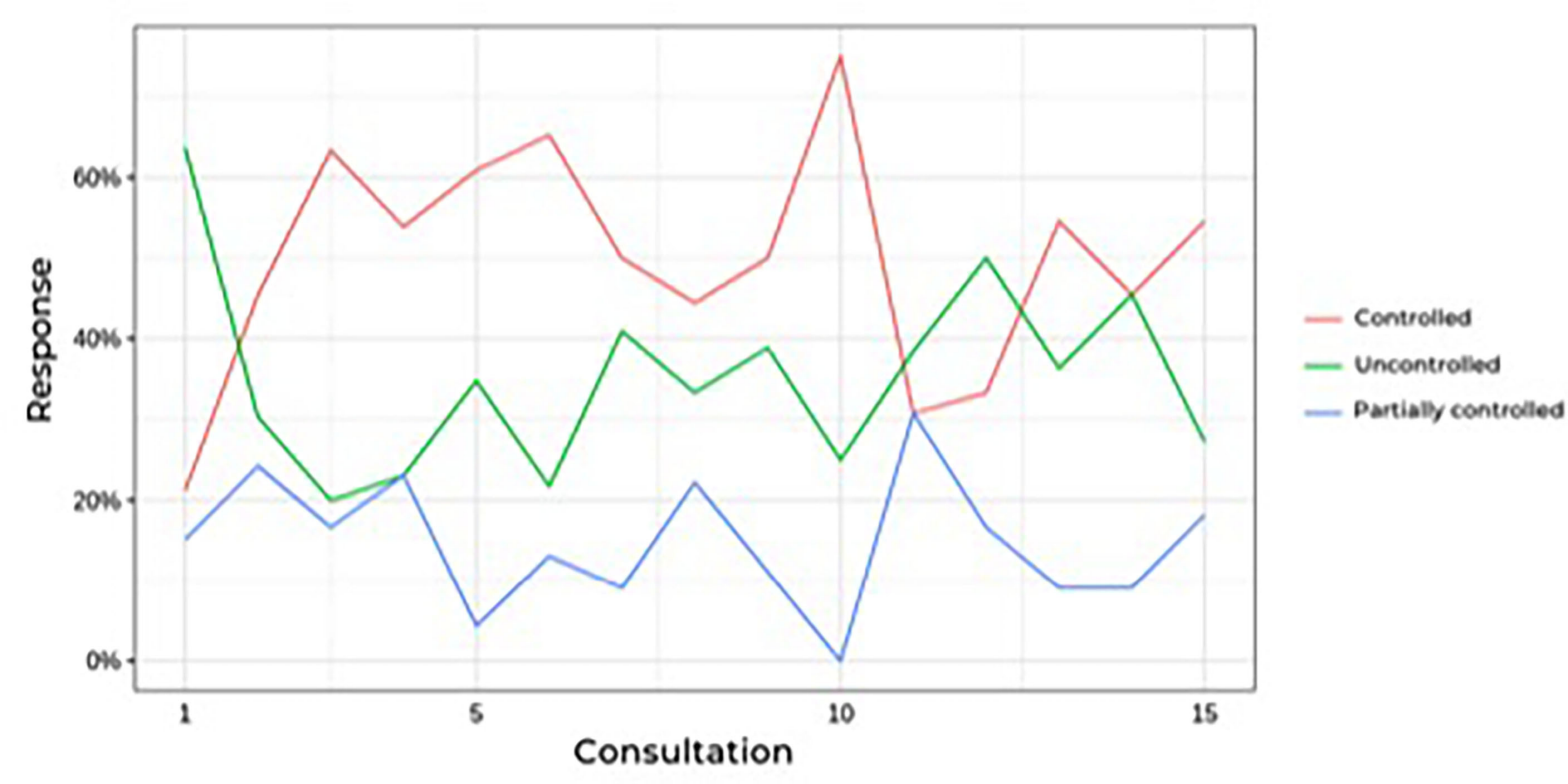
Prevalence of controlled hypercortisolism during follow-up of Cushing's disease patients treatesd with ketoconazole.

**Figure 2** shows that the prevalence of uncontrolled patients remained stable over time (approximately 30%) despite dose adjustments or association with other drugs, which led to no differences. When analyzing only the results of the last follow-up visit (eliminating fluctuations during follow-up), 22 patients had a complete response (66%), three patients had a partial response (9%), and eight patients had no response to ketoconazole treatment (24%), which includes patients who underwent radiotherapy during ketoconazole treatment.

**Figure 3:**
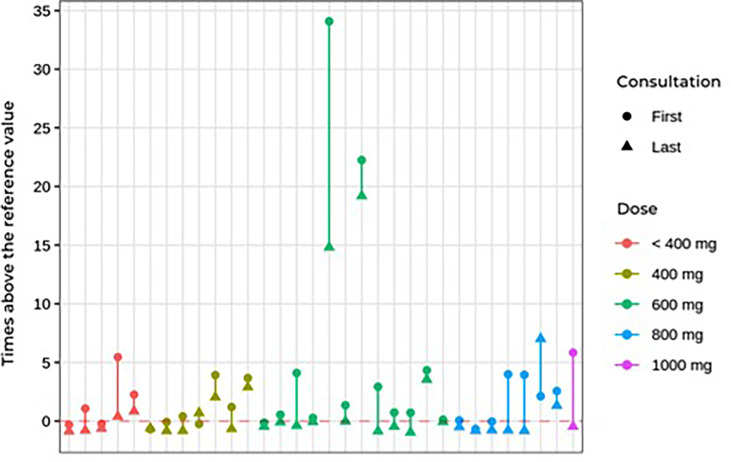
First and last consultation 24çhour UFC results vs. ketoconazole dosage in Cushing's disease patients.

During follow-up, no significant differences were found in blood pressure control or in dehydroepiandrosterone sulfate, cortisol, ACTH, or glucose levels. Worsening of hypertension control was observed in association with hypokalemia in some cases, as described in side effects. The ketoconazole doses ranged from 100 to 1,200 mg per day, and there were no significant dose or response differences between the groups (**Table 4**). **Figure 3** shows the patients, their dosages, and 24-h UFC control at the first and last consultation, showing a trend toward hypercortisolism reduction in approximately 70% of the cohort (25 of 33). Only four patients used doses lower than 300 mg at the end of follow-up. One of them used before TSS and suspended its use after surgery. One patient, who has already undergone radiotherapy, discontinued ketoconazole due to intolerance, despite adequate control of hypercortisolism. Another one, who had also undergone radiotherapy, was lost to follow-up when it was controlled using 100 mg daily, and one remained controlled using 200 mg, without previous radiotherapy.

**Table 4:** Final dose of ketoconazole used in patients with Cushing’s disease.

Final dose	Controlled/partially controlled	Uncontrolled
≤100 mg	1 (4%)	0
200 mg	2 (8%)	0
300 mg	0	1 (12.5%)
400 mg	6 (24%)	2 (25%)
600 mg	9 (36%)	3 (37.5%)
800 mg	6 (24%)	2 (25%)
1,000 mg	1 (4%)	0

### Side effects

Regarding adverse effects (**Table 5**), there was no significant difference between the controlled/partially controlled group and the uncontrolled group regarding liver enzyme changes or drug intolerance. Mild adverse effects, including nausea, vomiting, dizziness, and loss of appetite, occurred in 10 patients (30%). Only four patients had serious adverse effects that warranted discontinuing the medication. In two cases, ketoconazole was discontinued due to a significantly acute increase in liver enzymes (drug-induced hepatitis) during the use of 400 and 800 mg of ketoconazole. Non-significant elevation of transaminases (up to three times the normal value) was observed in three cases. A slight increase in gamma-glutamyltransferase occurred in six patients. In these nine patients with elevated liver markers, the daily dose ranged from 400 to 1,200 mg. None of those with mild increases in liver markers needed to discontinue ketoconazole.

**Table 5:** Adverse effects of ketoconazole in Cushing’s disease patients treated with ketoconazole.

	Controlled/partially controlled	Uncontrolled	*p*
AST	70 ± 230 (12–1,128)	59 ± 90 (15–244)	0.608
ALT	97 ± 341 (16–1,665)	112 ± 214 (17–550)	0.871
Intolerance (Y/N)	3/22 (12/88%)	1/7% (12.5–87.5%)	0.97

AST, aspartate transaminase; ALT, alanine aminotransferase.

One female patient developed pseudotumor cerebri syndrome, which was treated with acetazolamide. She did not need to discontinue ketoconazole, having used it for more than 10 years without new side effects and achieving complete control of hypercortisolism (24). Another patient became pregnant during follow-up while using the medication, but no maternal or fetal complications occurred (25).

Hypokalemia was also observed during follow-up. Twenty episodes of reduced potassium levels occurred in 10 patients over the course of treatment. Of these episodes, six occurred in controlled patients, three in partially controlled patients, and 11 in uncontrolled patients (**Table 6**). The hypokalemia was managed with spironolactone (25 to 100 mg) and oral potassium supplementation.

**Table 6:** Characteristics of Cushing’s disease patients who developed hypokalemia during ketoconazole treatment.

ID	Dose (mg)	HK episodes	K value	Disease control	BP control	Previous medications in use
9	1,200	7	3.3 3.3 3 2.8 2.8 2.8 3	No No No No Partial No No	High High Normal Normal Normal High Normal	HCTZ (suspension) enalapril, spironolactone, PPL, amlodipine, cabergoline, mitotane
15	600	1	3.2	Yes	Normal	HCTZ
16	1,000	1	2.8	Yes	High	Spironolactone, furosemide, enalapril, chlorthalidone, simvastatin
8	600	1	3.4	Yes	Normal	HCTZ
10	800	1	3.1	No	High	HCTZ
14	800	1	3.3	No	Normal	HCTZ and captopril
19	1,200	1	2.9	No	Unavailable	Amlodipine, atenolol, losartan, hydralazine, spironolactone
25	400	1	3.2	Partial	High	HCTZ
28	1,200	3	2.9 2.6 3.3	Partial Partial No	High High High	Losartan, chlorthalidone, spironolactone, hydralazine
27	400 400 600	3	3.1 3.4 3.4	Yes No Yes	Normal Normal Normal	Enalapril and HCTZ

HK, hypokalemia; HCTZ, hydrochlorothiazide; PPL, propranolol.

### Ketoconazole and associations

Of the patients who used an association of cabergoline and ketoconazole, one did so since the beginning of follow-up, while another nine were prescribed cabergoline during follow-up due to non-response to ketoconazole alone. Of these 10 patients, two did not start the medication due to problems in obtaining the drug. Thus, in two of the nine patients on the maximum tolerated dose of ketoconazole or who could not tolerate a higher dose due to hepatic enzymatic changes, 1.5–4.5 mg of cabergoline per week was associated. In patients not controlled with ketoconazole plus cabergoline, mitotane (two patients) or pasireotide (two patients) was added. Only two of nine patients responded to the combination of cabergoline and ketoconazole. Data on these associations are shown in **Table 7**.

**Table 7:** Effects of associating cabergoline with ketoconazole in Cushing’s disease patients.

ID	Sex	Adenoma	Maximum daily (mg) dose of ketoconazole	Maximum weekly dose of cabergoline	Control	Other medications	Side effects	Outcome
7	F	Macro	1,000	3.5 mg	No	Temozolomide and pasireotide	Gastrointestinal effects	RDT
9	F	Normal	1,000	3.5 mg	No	Mitotane	AI symptoms	Adrenalectomy
10	F	Macro	1,200	3 mg	Yes	–	–	Medications maintained
14	F	Macro	800	1.5 mg	No	–	Elevation of transaminases	RDT
15	F	Micro	1,200	3 mg	No	Mitotane	–	RDT
21	F	Normal	600	2 mg	Yes	–	Gastrointestinal symptoms	Adrenalectomy
26	F	Macro	400	2 mg	No	–	–	RDT
28	M	Macro	1,200	4.5 mg	No	Pasireotide	Hyperglycemia, hypogonadism, and hypokalemia	RDT

AI, adrenal insufficiency; ND, no data; RDT, radiotherapy.

Considering that one of the indications for the treatment of hypercortisolism may be complementary to radiotherapy, we analyzed the eight patients who underwent radiotherapy after transsphenoidal surgery. In these patients, doses of ketoconazole from 200 to 1,200 mg were used, and in six patients there was a normalization of the UFC in 1 to 60 months of treatment. Thus, the association of ketoconazole with radiotherapy was effective in normalizing the 24-h UFC in 75% of cases.

### Clinical follow-up

New therapeutic approaches were attempted in some patients during follow-up: radiotherapy (eight patients), new TSS (five patients), and bilateral adrenalectomy (four patients). At the end of this analysis, 11 patients remained on ketoconazole, all with controlled hypercortisolism. Among the 11 patients who were not fully controlled by the last visit, five were using ketoconazole as pre-TSS therapy and underwent TSS as soon as possible, while three others underwent radiotherapy and two underwent bilateral adrenalectomy. One patient was lost to follow-up.

## Discussion

According to the current consensus about CD, drug treatment should be reserved for patients without remission after TSS, those who cannot undergo surgical treatment, or those awaiting the effects of radiotherapy (4, 16). Drugs available in this context may act as adrenal steroidogenesis blockers (ketoconazole, osilodrostat, metyrapone, mitotane, levoketoconazole, and etomidate), in pituitary adenoma (somatostatinergic receptor ligands—pasireotide), dopamine receptor agonists (cabergoline), or glucocorticoid receptor blockers (mifepristone) (16, 26). Among these alternatives, the drug of choice still cannot be determined. Thus, the best option must be established individually, considering aspects such as remission potential, safety profile, availability, cost, *etc.* (16, 27, 28).

For over 30 years, ketoconazole has been prescribed off-label for CD patients with varied rates of remission of hypercortisolism, and it can be used in monotherapy or associated with other drugs (29, 30). The Brazilian public health system does not provide drugs for the treatment of CD, and among medications with a better profile for controlling hypercortisolism, such as osilodrostat, levoketoconazole, and pasireotide, only pasireotide has been approved by the national regulatory authority (ANVISA). Due to such pragmatic considerations, ketoconazole is among the most commonly used drugs in our health system, whether recently associated or not with cabergoline (7).

In this cohort, the most prevalent response type was complete (66%). Since 75% of the CD patients who used ketoconazole had a complete or partial response, there was a clear trend towards improvement in hypercortisolism. When only those who used ketoconazole post-TSS were evaluated, the rate of control increased to 76%. We found that patients with a higher initial 24-h UFC tended to have less control of excess cortisol, a difference that was not observed when analyzing ketoconazole dose or follow-up time.

In our series and at the prescribed doses, the combination of cabergoline and ketoconazole was not effective in the management of hypercortisolism since only two of nine patients (22%) had their 24-hour UFC normalized. However, it should be observed that this association was used in patients who had more severe CD and, consequently, were less likely to have a favorable response. The effects of cabergoline in CD patients remain controversial, although some studies have shown promising responses (31, 32).

Previous reviews found that the efficacy of ketoconazole for hypercortisolism control was quite heterogeneous, ranging from 14 to 100% in 99 patients (33, 34). Our cohort’s response rate was lower than that of Sonino *et al.* (89%) (20) but higher than that of a multicenter cohort by Castinetti *et al.* (approximately 50%) (14). Regarding other smaller series (35–37) our results reinforce some findings that demonstrate a percentage of control greater than 50% of the cases.

Our analyses showed a trend toward a response that continued, with some oscillations, over time. The rate of uncontrolled patients remained stable over time (approximately 30%), regardless of association with other drugs (cabergoline, mitotane, or pasireotide) or dose adjustments. Speculatively, it would appear that patients who respond to ketoconazole treatment would show some type of response as soon as therapy begins.

Our cohort has the longest follow-up time of any study on ketoconazole use in CD, nearly 15 years. Our results demonstrate that patients who benefit from ketoconazole (*i*.*e*., control of hypercortisolism and associated comorbidities) can safely use it for a long term since those who did not experience liver enzyme changes at the beginning of treatment also had no long-term changes.

Another relevant information for clinical practice is the result of treatment with ketoconazole associated with radiotherapy, which demonstrated normalizing the 24-h UFC in 75% of cases, a finding that reinforces the use of this therapeutic combination, especially in cases that are more resistant to different treatment modalities.

As described in the literature, adverse effects, such as nausea, vomiting, dizziness, headache, loss of appetite, and elevated transaminases, are relatively frequent (38). In our cohort, 10 patients (30%) had mild adverse effects, and four (12%) had more serious adverse effects requiring discontinuation. In other studies, up to 20% of patients required discontinuation due to side effects (14). We documented 20 episodes of hypokalemia during ketoconazole treatment, some with worsening blood pressure control. In most cases, hypokalemia has occurred in association with the use of diuretic drugs, which may have potentiated potassium spoliation, reinforcing the need of stringent surveillance in hypertensive Cushing’s disease patients using this combination. It can also result from the enzymatic blockade that could lead to the elevation of adrenal mineralocorticoid precursors (pex. deoxycorticosterone), with consequent sodium retention and worsening hypertension. Although it has not been analyzed in other series with ketoconazole, this side effect has been observed in patients who received other adrenal-blocking drugs, such as osilodrostat and metyrapone (16). This alteration seems to be transient in some patients; in our series, it was managed by suspending drugs that could worsen hypokalemia and introducing spironolactone and/or potassium supplementation. Hypokalemia may also result from continuing intense adrenal stimulation by ACTH and changes in the activity of the 11-beta-hydroxysteroid dehydrogenase enzyme, which increase the mineralocorticoid activity of cortisol, as observed in patients with severe hypercortisolism in uncontrolled CD (39). Hypogonadism occurred in one male patient. In two adolescent patients (one female and one male), hypercortisolism was effectively controlled without altering the progression of puberty. As described in other cohorts, this effect was expected due to the high doses, which block adrenal and testicular androgen production (20).

Thus, our findings confirm previous reports in the literature and add important information about the side effects and safety of long-term ketoconazole use in CD treatment. Our data reinforce the current recommendations about ketoconazole for recurrent cases or those refractory to surgery, including proper follow-up by an experienced team specializing in evaluating clinical and biochemical responses and potential adverse effects (7, 18, 40). Despite the severity of many of our CD patients, no ketoconazole-related death occurred during follow-up, including long-term observation. On the other hand, no patient progressed to definitive remission of hypercortisolism, even after many years of treatment with ketoconazole.

## Conclusions

In our cohort of patients, ketoconazole proved to be an effective and safe alternative for CD treatment, although it can produce side effects that require proper identification and management, allowing effective long-term treatment. We found side effects that have been rarely described in the literature, including hypokalemia and worsening hypertension, which require specific care and management. Thus, ketoconazole is an effective alternative for CD patients who cannot undergo surgery, who do not achieve remission after pituitary surgery, or who have recurrent hypercortisolism.

## Data availability statement

The raw data supporting the conclusions of this article will be made available by the authors without undue reservation.

## Ethics statement

The studies involving human participants were reviewed and approved by the Hospital de Clínicas de Porto Alegre Research Ethics Committee. Written informed consent for participation was not required for this study in accordance with the national legislation and the institutional requirements.

## Author contributions

CV and MAC created the research format. CV, RBM, and MCBC realized the search on medical records. CV performed the statistical analysis. MAC, ACVM, and TCR participated in the final data review and discussion. ACVM participated in the final data review and discussion as volunteer collaborator. All authors contributed to the article and approved the submitted version.

## Funding

This work was supported by the “Coordenação de Aperfeiçoamento de Pessoal de Nível Superior” (CAPES), Ministry of Health - Brazil, through a PhD scholarship; and the Research Incentive Fund (FIPE) of Hospital de Clínicas de Porto Alegre.

## Acknowledgments

The authors would like to thank the HCPA Research and Graduate Studies Group (GPPG) for the statistical technical support provided by Rogério Borges. We also thank the Research Incentive Fund of Hospital de Clínicas de Porto Alegre and Coordenação de Aperfeiçoamento de Pessoal de Nível Superior (CAPES), by funds applied. We also thank the Graduate Program in Endocrinology and Metabolism (PPGEndo UFRGS) for all the support in the preparation of this research.

## Conflict of interest

The authors declare that the research was conducted in the absence of any commercial or financial relationships that could be construed as a potential conflict of interest.

## Publisher’s note

All claims expressed in this article are solely those of the authors and do not necessarily represent those of their affiliated organizations, or those of the publisher, the editors and the reviewers. Any product that may be evaluated in this article, or claim that may be made by its manufacturer, is not guaranteed or endorsed by the publisher.
